# An open-label study of effects of acupuncture on chronic fatigue syndrome and idiopathic chronic fatigue: study protocol for a randomized controlled trial

**DOI:** 10.1186/1745-6215-14-147

**Published:** 2013-05-21

**Authors:** Jung-Eun Kim, Kwon-Eui Hong, Hyeong-Jun Kim, Jin-Bong Choi, Yong-Hyeon Baek, Byung-Kwan Seo, Sanghun Lee, Kyung-Won Kang, Min-Hee Lee, Joo-Hee Kim, Seunghoon Lee, So-Young Jung, Hee-Jung Jung, Mi-Suk Shin, Sun-Mi Choi

**Affiliations:** 1 Department of Medical Research, Korea Institute of Oriental Medicine, Acupuncture, Moxibustion & Meridian Research Group, 1672 Yuseongdae-ro, Yuseong-gu, Daejeon, South Korea; 2 Department of Acupuncture & Moxibustion, Daejeon University, 176-9 Daeheung-ro, Jung-gu, Daejeon, South Korea; 3 Department of Oriental Gynecology, Semyung University, 65 Semyung-ro, Jecheon, Chungbuk, South Korea; 4 Department of Oriental Rehabilitation Medicine, Dongshin University, 141 Weolsan-ro, Nam-gu, Gwangju, South Korea; 5 Department of Acupuncture & Moxibustion, College of Korean Medicine, Kyung Hee University, 892 Dongnam-ro, Gangdong-gu, Seoul, South Korea

**Keywords:** Acupuncture, Chronic fatigue, Randomized controlled trial

## Abstract

**Background:**

Even though chronic fatigue syndrome and idiopathic chronic fatigue are quite common, there are no clearly known causes. Most treatments are therefore symptomatic in nature, and chronic fatigue syndrome and idiopathic chronic fatigue patients are highly interested in using oriental medicine or complementary and alternative medicine treatment. Acupuncture, one of the major treatments used in oriental medicine, is effective in treating various diseases. This study will attempt to analyze the effectiveness and safety of acupuncture in the treatment of chronic fatigue by comparing the two treatment groups (body acupuncture, Sa-am acupuncture) and the control group (usual care).

**Methods/design:**

This study consists of a four-center, three-arm, randomized, controlled, and open-label trial. One hundred and fifty participants are randomly divided into treatment groups A and B and a control group. The treatment groups will receive acupuncture treatments either two or three times per week for a total of 10 sessions over a period of 4 weeks. The control group will not receive acupuncture treatments and will continue their usual care during this period. The primary outcome variable is the Fatigue Severity Scale, which will be utilized 5 weeks after randomization. Secondary outcome variables are the Fatigue Severity Scale at 13 weeks, a short form of the Stress Response Inventory, the Beck Depression Inventory, the Numeric Rating Scale, and the EuroQol-5 Dimension at 5 and 13 weeks after randomization.

**Discussion:**

This study will provide evidence with high external validity on the effectiveness and safety of acupuncture as a treatment for chronic fatigue syndrome and idiopathic chronic fatigue.

**Trial registration:**

Clinical Research Information Service KCT0000508

## Background

Fatigue is a subjective feeling of emptiness or a lack of power that can affect one’s daily life during or after work [1].

Fatigue can be classified according to the duration of symptoms. Fatigue is defined as transient fatigue when symptoms disappear within 1 month, continuous fatigue when symptoms last for longer than 1 month but less than 6 months, and chronic fatigue when symptoms last longer than 6 months [2]. Either a physical or a mental cause of chronic fatigue is medically describable for about two-thirds of patients. Medically indescribable cases of chronic fatigue can be classified into chronic fatigue syndrome (CFS) and idiopathic chronic fatigue (ICF) [3]. CFS is prevalent in 1% of the total population and ICF is prevalent in 10% [4]. The symptoms associated with a diagnosis of CFS include unexplained fatigue, lasting longer than 6 months, new or definite onset, fatigue that does not result from ongoing exertion and is not substantially alleviated by rest, and a substantial reduction in previous levels of occupational, educational, social, or personal activities. In addition to these symptoms, more than four of the following symptoms should be concurrently present over 6 months: impaired memory or concentration, sore throat, tender cervical or axillary lymph nodes, muscle pain, multi-joint pain, onset of new headaches, unrefreshing sleep, and post-exertion malaise. If a patient does not meet all the criteria for CFS described above, he/she is diagnosed with ICF [5].

Since there is no recognized treatment because the cause is not known, CFS and ICF patients are highly interested in treatment using oriental medicine or complementary and alternative medicine, and various approaches have been used [6].

Acupuncture is one of the most widely used oriental medicine treatments. The previous systematic reviews reported that many of the studies included in the reviews showed that acupuncture and moxibustion treatment were effective in treating chronic fatigue, but it is difficult to judge the level of evidence due to methodological flaws of the studies [7-11].

Aside from the aforementioned systematic reviews, three recently conducted randomized controlled trials used a non-meridian point group as a control group. In two of these trials, the treatment group showed a marked improvement compared with the control group [12,13], but no significant difference was observed in one study [14]. There has been no proven placebo for acupuncture. The non-meridian point groups in the aforementioned studies are clearly considered not to be physiologically inert. These studies may therefore have had limitations in separating the specific effects of acupuncture [15].

This study was planned so as to use two forms of acupuncture; the first form, body acupuncture, indicates acupuncture that is applied to the whole body, in comparison with microsystem acupuncture that is applied to the specific local areas such as scalp acupuncture and auricular acupuncture. Acupuncture has a history that is at least 2,000 years old and has been used widely in China, Korea, and Japan [16-18]; the second form, Sa-am acupuncture, is traditional Korean acupuncture, which was developed in the latter part of the Joseon Dynasty. Sa-am acupuncture is a school of acupuncture initiated by Sa-am, which is characterized by applying the five-phase theory and the mother–child reinforcement–reduction principle to the selection of points and needling manipulation [12,16]. The control group is the usual care group, and we will measure the overall effects of acupuncture.

The aim of this study is to provide grounds for the effectiveness and safety of acupuncture by comparing the two acupuncture treatment groups and the control group.

## Methods/design

### Design

A multicenter (four centers), randomized, controlled trial will be conducted at the Department of Oriental Rehabilitation Medicine at Gwangju Oriental Hospital of Dongshin University, the Department of Acupuncture and Moxibustion at Gangdong Oriental Hospital of Kyung Hee University, the Department of Oriental Gynecology at Jecheon Oriental Hospital of Semyung University, and the Acupuncture, Moxibustion, and Meridian Research Group of the Korea Institute of Oriental Medicine.

**Figure 1:**
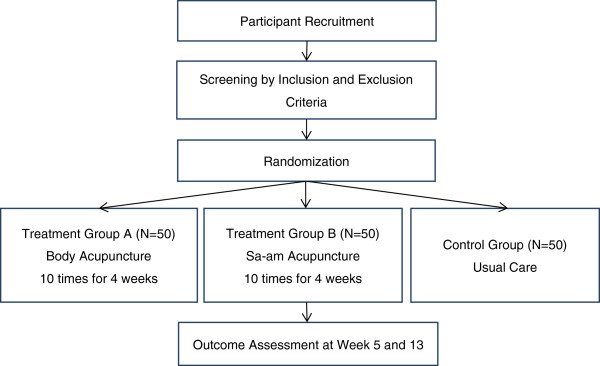
Study flow chart.

Participants who are considered suitable for the study will receive a total of 10 acupuncture treatments or usual care after they are randomly appointed to treatment group A (body acupuncture group), treatment group B (Sa-am acupuncture group), or the control group (usual care group). The treatment groups will receive the acupuncture treatments for 4 weeks, two to three times a week. This allows for a flexible treatment regime of once a week or four times a week up to each once. The total study period will be 13 weeks: a screening period of 1 week, a treatment period of 4 weeks, and a follow-up period of 8 weeks. The study flow is depicted in Figure 1.

### Randomization and allocation concealment

A total of 150 participants will be randomly allocated into treatment group A, treatment group B, or the control group. Dongshin, Kyung Hee, and Semyung University Hospitals will each recruit 45 patients, and the Korea Institute of Oriental Medicine will recruit 15 patients. To select the three groups, this study will use block stratified randomization that has four institutions as strata. A statistician will create the randomization list using a computer program (Strategic Applications Software, version 9.1.3; SAS Institute Inc., Cary, NC, USA). Randomly allocated envelopes will be sealed in opaque envelopes and delivered to each center and stored in double-locked cabinets. Random allocation will be performed at the second visit for participants who consented in written form and satisfied all selection standards. Before the implementation of random allocation, a clinical research coordinator at each clinical center will record the details of the newly included participant (name, date of birth, participant and center codes, date of inclusion) in case report forms and prepare a copy of the signed informed consent form. The researcher will open random allocation envelopes in order in front of participants and allocate them to each group. The random allocation envelops will be opened after participants complete baseline assessments. The practitioners and participants will get to know the allocated group according to the research design but the outcome assessors and data analysts will not know. The clinical research coordinator will provide participant identification codes and record them in case report form. The opened envelopes will be stored separately in double-locked cabinets.

### Blinding

This is an unblinded study.

### Ethics

Written consent will be obtained from each participant. This study’s protocol was approved by all relevant institutional review boards: Gwangju Oriental Hospital of Dongshin University, Gangdong Oriental Hospital of Kyung Hee University, Jecheon Oriental Hospital of Semyung University, and Daejeon Oriental Hospital of Daejeon University.

### Participants

This study will use the following the inclusion criteria: (1) males and females aged 19 to 65; (2) the presence of unexplained fatigue that is continuous and repetitive for more than 6 months; (3) the absence of abnormal findings in all of the following: blood pressure, complete blood count (hemoglobin, hematocrit, white blood cell, glucose), biochemical examination (aspartate aminotransferase, alanine aminotransferase, creatinine), electrolyte (sodium, potassium, chloride), thyroid function test (thyroid stimulating hormone, free thyroxine), pregnancy test (fertile woman), chest X-ray, and electrocardiogram; and abnormality criteria as follows: (a) diastolic blood pressure ≥90 mmHg (after putting the subjects at rest for more than 5 minutes, diastolic blood pressure is measured twice at an interval of 2 minutes or longer while the subjects are seated and the results are averaged); (b) hemoglobin <13 g/dl and hematocrit <38% for male adults, hemoglobin <11.5 g/dl and hematocrit <36% for female adults, white blood cell ≥11,000/mm^3^, random plasma glucose ≥200 mg/dl; (c) aspartate aminotransferase ≥50 IU/l and alanine aminotransferase ≥50 IU/l, creatinine ≥1.5 mg/dl; (d) sodium <135 mmol/l or ≥145 mmol/l, potassium <3.5 mmol/l or ≥5.5 mmol/l, chloride <97 mmol/l or ≥110 mmol/l; (e) thyroid stimulating hormone <0.35 mIU/ml or ≥5.50 mIU/ml, free thyroxine <0.89 ng/dl or ≥1.76 ng/dl; (f) urine human chorionic gonadotropin is (+); (g) lesions of pulmonary tuberculosis, other than inactive tuberculosis on a chest X-ray; (h) arrhythmia requiring treatment, ischemic heart disease, or cardiomegaly indicated from an electrocardiogram; (4) a Numeric Rating Scale [19] score ≥4 for the week prior to the screening visit; and (5) those who consent to participate in this trial and sign an informed consent statement after listening to a clear explanation of the purpose and characteristics of this clinical trial.

This study will use the following exclusion criteria: (1) the presence of the following conditions in the subject’s past history that might trigger chronic fatigue: (a) organic causes, such as acute or chronic liver disease (for example, hepatitis, liver cirrhosis), anemia, tuberculosis, chronic lung disease, cardiovascular disease (for example, heart failure, hypertension), endocrine/metabolic disease (for example, diabetes, thyroid gland disease, severe obesity), autoimmune disease (for example, rheumatoid arthritis, systemic lupus erythematosus, multiple sclerosis), malignant tumors, or infectious disease; and (b) psycho-social causes, such as depression, anxiety neurosis, recent severe stress, schizophrenia, alcoholism, or an eating disorder (anorexia nervosa, bulimia nervosa); (2) subjects who have taken the following drugs within the past 2 weeks: antihypertensive drugs, antidepressants, anti-anxiety agents, hypnotics, or antihistamines; (3) pregnant or breast-feeding women; (4) subjects who are participating in other clinical trials; (5) subjects who are overworked; (6) subjects who have experienced a hypersensitive reaction after acupuncture treatment (in the case of participants with an experience of acupuncture treatment); (7) subjects who are inmates at group facilities, such as social welfare institutions; (8) subjects who do not provide informed consent; and (9) others whose clinical trial conductors are considered inappropriate for participating in this trial [5,20].

We will recruit participants using advertisements in local newspapers, the websites of local universities, and posters displayed in hospitals. Respondents will go through telephone pre-screening led by the clinical trial coordinator to ensure they meet the inclusion criteria (1), (2), and (4). The next step will be for participants to visit their local clinical trial center and go through a more in-depth screening process to determine whether they satisfy the inclusion criteria or meet the exclusion criteria.

### Intervention

Treatment groups will receive acupuncture treatment a total of 10 times for 4 weeks, and the control group will continue receiving their usual care. Meridian points that were used in two pilot studies will be applied to the two treatment groups [12,18]. The practitioner who will be providing treatment is a doctor of Korean medicine with over 3 years of practical experience and will have one training session so that he/she will be able to treat accordingly. Disposable needles with a diameter of 0.25 mm and a length of 30 mm (Dongbang Acupuncture Incorporation, Gyeonggi-do, Korea) will be used.

#### Treatment group A (body acupuncture group)

The participants randomly assigned to treatment group A will be treated while they are in a prone position. At one acupuncture point on the head (GV20), the needle will be leaned anterior to the body and inserted 0.5 to 1.5 F-cun, unilaterally. At one acupuncture point on the neck (GB20), the needle will be inserted 0.3 to 1.0 F-cun in the direction of the opposite eye bilaterally. At six points in the back region (BL11, BL13, BL15, BL18, BL20, BL23), the needle will be leaned 45 to 90° towards the bottom and inserted 0.5 to 1.0 F-cun, bilaterally. The needles will be retained for 15 minutes without using the twirling method [18].

#### Treatment group B (Sa-am acupuncture group)

The participants appointed to treatment group B will be treated while they sit in a chair. Males will be treated on the left side and females will be treated on the right side.

The acupoints used for this treatment will all be unilateral. At LU8 in the wrist region, the needle will be leaned 45° downward and inserted 0.5 to 0.7 F-cun. At SP3 in the foot region, the needle will be leaned 45° upward and inserted 0.3 to 0.5 F-cun. At HT8 in the palm region, the needle will be leaned 45° downward and inserted 0.3 to 0.5 F-cun. At BL15 in the back region, the needle will be leaned 45 to 90° upward and inserted 0.5 to 1.0 F-cun. Finally, at CV6 in the abdominal region, the needle will be leaned 45° downward and inserted 0.5 to 1.0 F-cun.

The needles will be retained for 15 minutes. The needles will be twirled nine times for LU8, SP3, and HT8, and twirled six times for BL15 and CV6 [12,21].

### Control group (usual care group)

Participants in the control group will not receive acupuncture treatment A (body acupuncture) or B (Sa-am acupuncture) but will be able to receive the necessary individualized usual care except acupuncture treatment for chronic fatigue.

### Permitted and prohibited concomitant treatments

Participants in both treatment groups and the control group will be allowed to use any other form of treatment, including acupuncture, moxibustion, herbal medicine, physical treatment, conventional medication, over-the-counter drugs, supplements, and exercise, with the exception of acupuncture treatment for chronic fatigue. Educational materials explaining chronic fatigue will be given to participants in all three of the groups.

### Collection of baseline information

This study will collect information on the participants’ sex, age, height, weight, body mass index, education degree, job, marital status, regular eating habits, frequency of exercise, history of smoking, motivation for participating, and past health history related to chronic fatigue.

### Outcome measures

#### Primary

The Fatigue Severity Scale (FSS) will be used as the primary outcome measurement. The FSS uses a series of nine questions to evaluate the participants’ level of fatigue over the previous week applying a rating scale between 1 and 7. The final FSS score represents the average level of fatigue, and higher scores mean more fatigue [22].

#### Secondary

A short form of the Stress Response Inventory evaluates the participants’ stress reactions using the revised Stress Response Inventory, which uses a total of 22 questions covering the following three categories: somatization (nine items), depression (eight items), and anger (five items). For each of the questions, participants are asked to indicate the degree to which they have experienced stress over the prior week. The analysis is conducted by adding the scores within each category and then determining the total score [23].

The Beck Depression Inventory consists of 21 questions, including recognition, emotional, motivational, and physical symptoms of depression. Each category has four items that describe the participants’ level of depression. Scores range from 0 to 3 for each choice (items 1 to 4), and the scores are summed for analysis [24].

The Numeric Rating Scale uses a horizontal line scaled ranging from 0 to 10 and participants can choose their level of fatigue (0 = ‘no fatigue’ and 10 = ‘most severe level of fatigue one can imagine’) [18].

The EuroQol-5 Dimension evaluates health-related quality of life and consists of the EuroQol-5 Dimension descriptive system and the EuroQol-5 Dimension Visual Analogue Scale. The EuroQol-5 Dimension descriptive system consists of five dimensions, including mobility, self-care, usual activities, pain/discomfort, and anxiety/depression. Each of these is further evaluated into three levels. The EuroQol-5 Dimension Visual Analogue Scale indicates the participants’ health condition on a scale ranging from 0 to 100 [25,26].

### Other assessment measures

The treatment expectancy of each subject [27], as well as whether or not the subject satisfies the diagnostic criteria for CFS [2,5], will also be assessed.

The detailed outcome assessment time points are provided in Table 1.

**Table 1:** Schedule for treatment and outcome assessment

**Period**	**Screening**	**Treatment**										**Follow**-**up**	
Visit	1	2	3	4	5	6	7	8	9	10	11	12	13
Week		1~4										5	13
Informed consent	•												
Demographic characteristics	•												
Medical history	•												
Laboratory test, chest X-ray, electrocardiogram, pregnancy test	•												
Inclusion/exclusion criteria	•												
Numeric rating scale	•	○										•	•
Vital signs	•	•	○	○	○	○	○	○	○	○	○	•	•
Change of medical history		•	○	○	○	○	○	○	○	○	○	•	•
Random allocation		•											
Treatment expectancy questionnaire		•											
Chronic fatigue syndrome questionnaire		•											
Fatigue Severity Scale	•	○										•	•
A short form of Stress Response Inventory	•	○										•	•
Beck Depression Inventory	•	○										•	•
EuroQol-5 Dimension	•	○										•	•
Acupuncture treatment		○	○	○	○	○	○	○	○	○	○		
Safety assessment		•	○	○	○	○	○	○	○	○	○	•	•

•, all groups; ○, treatment groups.

### Hypothesis and sample size

The following two hypotheses are related to the differences between the treatment groups and the control group:

$$H_{0}:\mu_{1}=\mu_{3,}\;\mu_{2}=\mu_{3}$$

$$H_{1}:\mu_{1}\neq \mu_{3}\;\mathrm{or}\;\mu_{2}\neq \mu_{3}$$

where μ_1_ is the FSS mean score for treatment group A, 5 weeks after random allocation; μ_2_ is the FSS mean score for treatment group B, 5 weeks after random allocation; and μ_3_ is the FSS mean score for control group, 5 weeks after random allocation.

Referring to the previous study [28], the researchers expect that the difference between treatment group A and the control group will be smaller than the difference between treatment group B and the control group. This study therefore calculated the sample size taking into consideration the difference between treatment group A and the control group while applying the Bonferroni method, which is conservative among multiple comparison methods.

In the present study, changes from baseline in FSS mean score in the control group were calculated under the assumption that they would approximate 5% of changes in FSS mean score for treatment group A. The final calculated difference in changes in FSS mean score between the two groups was 0.915 with a standard deviation of 1.1479 [12,29].

Using the mean comparison method, this study will use an α value of 5%, a power of 90%, and the two-sided test:

$$\begin{array}{ll} \;n & =\frac{2\times {\left(Z_{\alpha /4}+Z_{\beta }\right)}^{2}\times \sigma^{2}}{\delta^{2}}\\ & =\frac{2\times {\left(2.2414+1.282\right)}^{2}\times 1.1479^{2}}{0,915^{2}}=39.08\approx 40 \end{array}$$

When the same number of patients is allocated for treatment groups A and B and the control group, each group has 40 patients. Considering a 20% dropout rate, each group will require 50 initial participants.

### Statistical methods

The analysis set consists of a full analysis set and a per-protocol set.

The full analysis set represents the set of subjects that is as close as possible to the ideal implied by the intention-to-treat principle.

The per-protocol set is the set of data generated by the subset of subjects who comply with the protocol sufficiently enough to ensure that these data will be likely to exhibit the effects of treatment, according to the underlying scientific model. Compliance covers such considerations as exposure to acupuncture treatment eight or more times, the availability of measurements, and the absence of major protocol violations [30].

Both sets will be analyzed and the full analysis set will be considered to be the primary analysis.

Of the sociodemographic characteristics and treatment expectancy, continuous data will provide the mean and standard deviation while the categorical data will provide the frequency and percentage.

For the treatment expectancy questionnaire, this study will use analysis of variance. When there is a significant between-group difference, we will add treatment expectancy as a covariate and use analysis of covariance for a primary outcome variable and compare it with the analysis results that do not use treatment expectancy as a covariate.

The CFS questionnaire will provide the frequencies and percentages of participants who adequately meet the CFS diagnosis criteria.

For the group comparisons of these variables, this study will use one-way analysis of variance when the continuous data satisfy a normal distribution or will use the Kruskal–Wallis test when these data do not follow a normal distribution. For categorical data, a chi-squared test will be used.

The primary and secondary outcomes 5 and 13 weeks after random allocation are the dependent variables; the baseline observed value is the covariate, and the allocated group and the institutions are factors; this study will use analysis of covariance. Testing of the hypotheses will be done using an α value of 5% and a power of 90%. When analysis of covariance acquires significant results, then a multiple comparison will be conducted to see which groups show differences.

Missing data will be processed with mixed model for repeated measurements method.

### Data and safety monitoring

Regular monitoring will be conducted for quality control. Monitoring confirms whether the practical research-related data are correct compared with the source document as well as whether the research follows the approved protocol.

Adverse events indicate undesirable and unintentional signs, symptoms, or diseases that appear after the practical experiment, and they do not have to be causally related to the treatment. Safety will be evaluated based on the frequency, severity, and symptoms of the adverse event.

## Discussion

This study is designed as a pragmatic clinical trial to more closely reflect the effects of everyday treatment of acupuncture [31]. The results of this study are expected to provide evidence, with high external validity, for the effectiveness and safety of two common forms of acupuncture treatment on chronic fatigue patients.

In this study, the meridian points that will be used on the participants in treatment group A are intended to relieve the mind, clear the spirit, tranquilize the body to free it from tension, and regulate the functions of internal organs [18].

Sa-am acupuncture treatment is largely divided into three types: fixed pattern, transformed pattern and experienced prescription. In this study, the two latter types were applied to the participants in the treatment group B [12,32].

## Trial status

The present study is currently recruiting participants.

## Abbreviations

CFS: Chronic fatigue syndrome; FSS: Fatigue Severity Scale; ICF: Idiopathic chronic fatigue.

## Competing interests

The authors declare that they have no competing interests.

## Authors’ contributions

K-EH, H-JK, J-BC, Y-HB, B-KS and J-EK drafted the protocol, and J-EK wrote the final manuscript. SaL, K-WK and M-HL conducted statistical design of the trial and wrote part of the statistical methods. J-HK, ShL, S-YJ, H-JJ and M-SS provided technical advice and made critical revisions. S-MC participated in the trial design as the principal investigator. All authors read and approved the final manuscript.
